# The public-private decision for alcohol retail systems: Examining the economic, health, and social impacts of alternative systems in Finland

**DOI:** 10.1177/14550725231160335

**Published:** 2023-03-15

**Authors:** Adam Sherk, Tim Stockwell, Justin Sorge, Samuel Churchill, Colin Angus, Tanya Chikritzhs, John Holmes, Petra Meier, Timothy S. Naimi, Thor Norström, Mats Ramstedt, Jussi Simpura

**Affiliations:** 8205 University of Victoria, Canada; 8205 University of Victoria, Canada; 8205 University of Victoria, Canada; 8205 University of Victoria, Canada; 7315 University of Sheffield, UK; Curtin University, Australia; 7315 University of Sheffield, UK; 3526 University of Glasgow, UK; 8205 University of Victoria, Canada; Stockholm University, Sweden; 7641 The Swedish Council for Information on Alcohol and Other Drugs, Sweden; 577176 Finnish Foundation for Alcohol Studies, Finland

**Keywords:** alcohol-caused harms, alcohol policy, alcohol retail systems, alcohol use, modelling study

## Abstract

**Background:** Organising alcohol retail systems with more or less public ownership has implications for health and the economy. The aim of the present study was to estimate the economic, health, and social impacts of alcohol use in Finland in 2018 (baseline), and in two alternative scenarios in which current partial public ownership of alcohol retail sales is either increased or fully privatised. **Methods:** Baseline alcohol-attributable harms and costs were estimated across five categories of death, disability, and criminal justice. Two alternate alcohol retail systems were defined as privately owned stores selling: (1) only low strength alcoholic beverages (public ownership scenario, similar to Sweden); or (2) all beverages (private ownership scenario). Policy analyses were conducted to estimate changes in alcohol use per capita. Health and economic impacts were modelled using administrative data and epidemiological modelling. **Results:** In Finland in 2018, alcohol use was estimated to be responsible for €1.51 billion (95% Uncertainty Estimates: €1.43 billion, €1.58 billion) in social cost, 3,846 deaths, and 270,652 criminal justice events. In the public ownership scenario, it was estimated that alcohol use would decline by 15.8% (11.8%, 19.7%) and social cost by €384.3 million (€189.5 million, €559.2 million). Full privatisation was associated with an increase in alcohol use of 9.0% (6.2%, 11.8%) and an increase in social cost of €289.7 million (€140.8 million, €439.5 million). **Conclusion:** The outcome from applying a novel analytical approach suggests that more public ownership of the alcohol retail system may lead to significant decreases in alcohol-caused death, disability, crime, and social costs. Conversely, full privatisation of the ownership model would lead to increased harm and costs.

Organising alcohol retail sales as systems with more or less public ownership has implications for public health (Babor et al., 2010; Hahn et al., 2012). The repeal of prohibition laws in the early 20th century saw the creation of public (i.e., government-owned) retail systems in Northern Europe, USA, Canada, and India (Room & Örnberg, 2019; Stockwell et al., 2018). Over time, these systems of retail have tended towards increased private ownership: either completely, as in many U.S. states and one Canadian province, or through the gradual increase of private market share due to changes such as the privatisation of beer and/or wine sales (Hahn et al., 2012). In these latter cases, the jurisdiction maintains public retail of higher strength products, e.g., Alabama and Idaho have public retail monopolies of spirts only (Hahn et al., 2012).

Systematic studies have shown that moving from retail systems with more public ownership to one with more private ownership may have consequences for public health. Hahn et al. (2012) conducted a review of retail system privatisations and identified 12 events wherein the sale of at least one beverage category was privatised. A median increase of 44.4% in per capita sale volume of the privatised beverage and a median decrease of 2.2% in the non-privatised beverages was identified. The authors conclude that privatisations led to an overall increase in alcohol use per capita, which is an important predictor of alcohol-caused harms (Rossow & Mäkelä, 2021). Studies on the partial privatisation of British Columbia's alcohol retail system reported increased hospital admissions (Stockwell et al., 2013) and deaths (Stockwell et al., 2011). A study of the re-monopolisation of medium strength beer sales in Sweden in 1977 reported a decrease in hospitalisations for alcohol use disorders (Ramstedt, 2002).

Finland currently maintains a public retail system, called Alko, for all alcoholic beverages above 5.5% alcohol by volume (ABV), while beverages with 5.5% ABV and below are sold at around 5,000 further retailers, mainly grocery stores. At the time of study in 2018, Alko operated 357 retail stores and its market share was approximately 38% of Finland's alcohol market, while 46% was sold in grocery stores and the remaining 16% was sold in bars and restaurants (Stockwell et al., 2019). Finland has a long history with alcohol retail system change. Alko was established after the repeal of prohibition in 1932. An important change occurred in 1969, when the sale of alcohol below 4.7% ABV was privatised (i.e., sold in grocery stores); in the two years after this change Finland experienced a nearly 50% increase in alcohol use per person (Mäkelä, 2002). In 2017, Finland passed a new Alcohol Act, with the provision of increasing the ABV limit of privatised beverages to 5.5%, thus permitting sales of stronger beers and alcopops in grocery stores (Stockwell et al., 2019). It is in this context that the present study is set.

In the present study, we estimated the potential economic and health impacts of two alternative policy scenarios in which public ownership of the alcohol retail system is either increased or eliminated. Previous modelling suggested that privatising Sweden's retail monopoly would increase alcohol use per capita and alcohol-caused deaths, hospital stays, assaults, and sick days (Norström et al., 2010; Stockwell et al., 2018); it may therefore be reasonable to hypothesise that increasing alcohol retail privatisation in Finland would lead to economic and health consequences. The aim of the present study was to create evidence, in the Finnish context, towards the effect of alcohol retail re-monopolisation and privatisation on alcohol use, alcohol-caused harms, and the associated economic impacts. The extension of outcomes to include criminal justice events, long-term disability cases, and economic costing may provide added evidence to Finnish policymakers wishing to address the harms and costs caused by alcohol in Finnish society.

## Methods

### Overview of the study method

We first estimated baseline harms and costs attributable to alcohol use in Finland in 2018 using a comparative risk assessment methodology (GBD 2016 Alcohol Collaborators, 2018; World Health Organization, 2018) and then compared these to estimates made under two alternative policy scenarios. The chosen scenarios were as follows: (1) increased public ownership of the alcohol retail system and regulation (similar to neighbouring Sweden); and (2) full privatisation of the alcohol retail system.

The alcohol-attributable harms and related costs estimated in each scenario were deaths, hospital stays, long-term disabilities, and three criminal justice outcomes (police-reported crime events, court cases, and corrections cases). We used the human capital approach (Sorge et al., 2019) to estimate the lost productivity costs of all potential years of productive life lost due to death and the lost productivity costs of long-term disability.

Key evidence-based alcohol policies, which would be expected to change in alternative alcohol retail scenarios were identified as pricing (mean and minimum prices) availability (hours and days of sale), based on previous policy modelling studies (Norström et al., 2010; Stockwell et al., 2017) and comprehensive reviews (Babor et al., 2010). For each scenario, we used key informants and local data to estimate the extent of these policy changes. We applied relevant published elasticities to estimate impacts on alcohol use per capita of these specific policy changes.

The International Model of Alcohol Harms and Policies (InterMAHP) (Sherk et al., 2017; Sherk, Stockwell, April, et al., 2020; Sherk, Stockwell, Rehm, et al., 2020), an open access web-based resource, was used to apply alcohol-attributable fractions to Finnish data on hospital stays and deaths in each scenario.

### Data sources and methods for estimating baseline harms and costs, by outcome category

Five categories of harm and costs from alcohol were estimated for men and women aged 15–24, 25–34, 35–64, and 65+ years as follows: (1) mortality; (2) potential years of productive life lost, (3) long-term disability cases, (4) hospital stays, and (5) criminal justice events, each described below.

#### Alcohol use per capita, the prevalence of alcohol use, and the distribution of drinking

We used Statistics Finland reported alcohol use per capita in litres of ethanol for the population aged 15+ years in 2018 (Statistics Finland., 2020c). Prevalence of current drinking, binge drinking, former drinking, and mean daily consumption were drawn from the 2016 Finnish Drinking Habits survey, which was received via request from the Finnish Institute for Health and Welfare (THL) and at the time of the study was the most recent available Finnish survey on alcohol use. The distribution of different levels of average daily consumption in the population was assumed to follow a Gamma distribution following Kehoe et al. (2012). InterMAHP was used to apply established risk functions for alcohol-attributable diseases and injuries (Sherk et al., 2017, 2020).

#### Hospital admissions and costs

Hospital stays caused by alcohol-related conditions for 2016 were received from THL defined by previously established ICD10 codes (Stockwell et al., 2019). Data were projected to 2018 using Statistics Finland's population change adjustments to match the latest available total consumption data (Statistics Finland, 2018e). To estimate alcohol-attributable fractions for partially attributable diseases, InterMAHP applies systematic review evidence on the risk of morbidity from these diseases at different levels of average daily alcohol use to observed disease incidence and alcohol use data. THL provided the average cost of a hospital stay, grouped by ICD10 chapter, which was applied to the alcohol-attributable hospital stays to arrive at economic costs (see Appendix A1).

#### Mortality, potential years of productive life lost, and economic loss of production

Mortality data were taken from Statistics Finland (Statistics Finland, 2020a, 2020b), grouped by major types of underlying cause of death (e.g., cancer, heart disease, injury). Alcohol-attributable fractions for disease and injury mortality were estimated using InterMAHP. For each death, potential years of productive life lost were calculated from age of death data for those who died before the age of 65 years, the assumed mean age of retirement. For deaths below 15 years of age, we assumed 50 years of lost production. These estimates were adjusted for workforce participation (Statistics Finland, 2018d). The economic cost of the resulting loss of production was estimated based on the Finnish average wage (Statistics Finland, 2018c). This is known as the human capital approach and is recommended by international guidelines for substance use cost studies (Single et al., 2003) and follows methods in a Canadian cost of substance use study (Canadian Substance Use Costs and Harms Scientific Working Group, 2018; Sorge et al., 2019).

#### Long-term disability cases

Long-term disability pension cases for 2017, by ICD10 chapter groupings, were obtained from the Finnish agency that manages long-term disability benefits, Kansaneläkelaitos (Kela; (KELA, 2018)) and population-adjusted to 2018 (Statistics Finland, 2018e). Aggregate alcohol-attributable fractions by age and sex for each ICD10 chapter calculated above for hospital stays were applied to estimated productive years living with a disability. As in 2.3, the estimated potential years of productive life lost were adjusted by workforce participation (Statistics Finland, 2018d) and the average wage (Statistics Finland, 2018c) was used to estimate costs of lost production.

#### Criminal justice: Policing, courts, and corrections

Police-reported crime event data, court cases, corrections cases, and national expenditures for 2016 were obtained from Statistics Finland (Statistics Finland, 2018a, 2018b). Crime events were divided into four categories: homicide; other violent crimes; non-violent crime; and alcohol-defined crimes (such as drink driving). The percentage of each crime type estimated to be caused by alcohol was calculated using Finnish data where available, and international data otherwise. See Appendix A2 for the detailed methodology.

### Policy analysis: Methods for estimating the impact of alcohol retail system scenarios on consumption

#### Alcohol retail system scenarios: Baseline, more public, or more private

We evaluated two alternative policy scenarios. In scenario 1 (S1: Public Ownership), Finland extends its publicly owned state retail monopoly to all alcoholic beverages of 3.5% ABV with reduced hours and days of sale, like neighbouring Sweden. In scenario 2 (S2: Private Ownership), Alko is disbanded and all types of alcohol can be sold in grocery stores.

#### Alcohol policy changes and impacts on alcohol use

Table 1 presents the baseline values of key alcohol pricing and availability measures and estimates of how these would change in each alternative scenario, both for products above and below 5.5% ABV. We detail below how these changes and their resulting impacts on alcohol use per capita were estimated.

**Table 1.** Estimated baseline levels and changes under two alcohol retail system scenarios of five key alcohol policies, by two alcohol strength bands, in Finland, 2018.

ABV strength band	Policy	Baseline Finland in 2018	Scenario 1: Public ownership (similar to Sweden)	Scenario 2: Private ownership (all alcohol sold in grocery stores)
Beer, cider, and long drinks >3.5% to 5.5% ABV	Mean price level	100^a^	106.46 (+6.46%)	100
	Cheapest price level	100^a^	128.09 (+28.09%)	100
	Sunday trading?	Yes	No	Yes
	Mon–Sat hours	72 hours / week	50 hours / week	72 hours / week
	Outlet density	5,165 stores	251 stores	4,808 stores
Beer, cider, and long drinks >5.5%, and all wine and spirits	Mean price level	100^a^	100	93.93 (−6.07%)
	Cheapest price level	100^a^	100	78.07 (−21.93%)
	Sunday trading?	No	No	Yes
	Mon–Sat hours	69 h / week	50 h / week	72 h / week
	Outlet density	357 stores	251 stores	4,808 stores

^a^The baseline average price and baseline cheapest price levels were defined as 100.

##### The average price of alcohol

We compared retail alcohol prices between Alko stores and two major grocery chains (S and K) in September 2018. Products were directly matched (by brand, size, and strength) and results were weighted by sales volume. Mean prices were 6.46% higher in Alko stores (see Table 1). We assumed these price differences would also apply for higher strength products not currently permitted for sale in grocery stores but would be in scenario 2. We assumed prices would otherwise stay the same if the permitted places where they could be sold did not change. To carry this price change through to an estimated change in alcohol use per capita, we applied price elasticities of alcohol demand of −0.39 for beer, −0.95 for wine, and −0.46 for spirits from a recent Finnish study (Ollikainen, 2016). These elasticities were within ranges provided by previous Finnish studies (Leppänen & Österberg, 2002; Vihmo, 2006) and international reviews (Wagenaar et al., 2009).

##### The minimum price of alcohol

A comparison was also conducted on the prices of the five cheapest beverages available for sale in Alko versus the S and K grocery stores for each beverage type. Products were weighted by sales volume and percentage differences were applied by scenario and strength band. As shown in Table 1, mean weighted prices were 21.93% lower overall in grocery stores. In the absence of Finnish estimates for minimum price elasticities, we applied a minimum price elasticity of −0.34 from a Canadian study (Stockwell et al., 2012).

##### Sunday sales

In 2018, Alko was closed on Sundays, while grocery stores were open every day. It was assumed there would be Sunday trading in scenario 2 but not in scenario 1. Based on a recent systematic review (Sherk et al., 2018), adding Sunday sales was assumed to increase APC by 3.4%.

##### Hours of alcohol sales

To avoid double counting the effects of both days and hours of sale, we did not count additional hours on Sunday. In 2018, Alko stores were open 69 hours while grocery stores were open 72 hours from Mon-Sat. In scenario 1, we assume the same opening hours as in Sweden at the time, i.e., 50 h on Mon-Sat (Stockwell et al., 2019). In the absence of studies quantifying impacts on APC of changes in hours of sale, the impact of one extra hour per week was assumed to be one-eight of a full day effect (Sherk et al., 2018).

##### The density of alcohol outlets

Table 1 shows the number of publicly and privately owned stores selling alcohol in Finland in September 2018 according to beverage type and strength, and how these would change if Finland matched store density in Sweden (scenario 1) or disbanded Alko (scenario 2). Sherk et al. (2018) identified four studies examining the relationship between density and APC (Brenner et al., 2015; Stockwell et al., 2009; Trolldal, 2005; Xie et al., 2000); however, these used heterogeneous density measures and the magnitude of density changes in our scenarios (−34% and >1,000%) were well beyond ranges explored by the studies. Available Canadian data were reanalysed (Stockwell et al., 2009) to estimate a decay function for the impact of increasing density on alcohol-attributable hospital admissions. This methodology is detailed elsewhere (Stockwell et al., 2017, p. 68). The resulting decay function was applied to estimate the impact of the very large density increase resulting from scenario 2.

#### Estimating the impact of these policy changes on per capita alcohol use

The collective effect of these policy-specific estimates was assumed to be additive, as in a previous policy modelling study (Stockwell et al., 2018). An elasticity of −0.97 was calculated for the effects of travellers’ imports on total recorded consumption Finland based on monthly data provided by Alko (see Table 2).

**Table 2.** Estimated alcohol use per capita changes under two alcohol retail system scenarios of five key alcohol policies, by two alcohol strength bands, in Finland, 2018.

ABV strength band	Policy	Scenario 1: Public ownership (similar to Sweden) Estimate (%) (95% UE)	Scenario 2: Private ownership (all alcohol sold in grocery stores) Estimate (%) (95% UE)
Beer, cider, and long drinks >3.5% to 5.5% ABV	Mean price level	−3.51 (−6.07, −0.82)	0
	Cheapest price level	−8.70 (−14.4, −2.24)	0
	Sunday trading?	−2.12 (−4.23, +0.03)	0
	Mon–Sat hours	−5.91 (−9.67, −3.15)	0
	Outlet density	−14.37 (−15.87, −12.83)	−0.17 (−0.27, −0.10)
	Subtotal	*−34.73* (−43.76, −24.96)	*−0.17* (−0.27, −0.1)
Beer, cider, and long drinks >5.5% ABV, and all wine and spirits	Mean price level	0	+3.76 (−1.95, +9.69)
	Cheapest price level	0	+7.17 (+3.2, +11.12)
	Sunday trading?	0	+2.13 (+0, +4.31)
	Mon–Sat hours	−5.49 (−8.05, −3.14)	+1.13 (+0.9, +1.37)
	Outlet density	−2.06 (−2.58, −1.55)	+16.03 (+14.07, +18)
	Subtotal	*−7.53* (−10.17, −5.11)	+*30.28* (+20.82, +39.88)
Weighted total across ABV bands		*−22.94* (−28.62, −17.1)	+*13.02* (+8.93, +17.17)
Cross-border effects		+7.15 (+5.33, +8.93)	−4.06 (−5.35, −2.79)
Final estimated change in alcohol use per capita		***−15.78 (−19.70, −11.77)***	***+8.96 (+6.15, +11.82)***

*Note.* UE = uncertainty estimate.

#### Estimating uncertainty intervals

We collected published standard errors and confidence intervals around all above consumption parameters. 95% confidence intervals (CIs) were estimated for the mean effects of policy changes on alcohol use per capita using a probability sensitivity analysis. This involved taking 10,000 random draws from a probability distribution around each estimate.

### Alcohol harms and costs analyses: Methods for estimating impacts of consumption changes on alcohol-attributable harms and costs

InterMAHP was used to estimate changes in alcohol-attributable fractions, by health condition, gender, and age group, which would occur under scenarios 1 and 2. These methods are detailed elsewhere (Sherk et al., 2017; Sherk, Stockwell, April, et al., 2020; Sherk, Stockwell, Rehm, et al., 2020, Stockwell et al., 2019) and important points are described below.

Changes to the shape of the Gamma distribution (Kehoe et al., 2012) representing the continuous prevalence distribution of daily average alcohol use were modelled using InterMAHP and estimated alcohol use per capita changes in each scenario. The prevalence of former and current drinkers (in the past year) was assumed to be unchanged as in previous studies (Sherk, Stockwell, April, et al., 2020, Stockwell et al., 2018), and the prevalence of binge drinkers was adjusted proportionally as defined elsewhere (Sherk, Stockwell, April, et al., 2020, p. 3). Changes to wholly attributable conditions were estimated mathematically as defined expressly for this purpose (Churchill et al., 2020).

For violent crime, we used a Finnish estimate where, for every 1 litre per year increase in APC, there was a resultant 11.7% increase in violent crime events (Norström & Ramstedt, 2020).

## Results

The estimated effects of individual policy changes on alcohol use per capita (APC), as well as subtotal estimates for the two ABV strength bands and weighted overall totals, are shown in Table 2. A 15.78% reduction in APC was calculated for scenario 1 and an 8.96% increase in APC for scenario 2. In each scenario, the largest components of these changes in total alcohol use were the effect of changing store density followed by the effect of changing the prices of the cheapest alcohol. All estimated impacts on alcohol-attributable harms and costs estimated for Finland in 2018 and in the two alternative policy scenarios are summarised in Table 3, with more detailed breakdowns in Tables S1–S4.

**Table 3.** Estimated levels at baseline and changes under two alcohol retail system scenarios of alcohol-attributable harms and costs in Finland, 2018.

	Category	Baseline Finland in 2018	Scenario 1: Public ownership (similar to Sweden)		Scenario 2: Private ownership (all alcohol sold in grocery stores)	
		Estimate (95% UE)	Estimated change (95% UE)	Percentage change (95% UE)	Estimated change (95% UE)	Percentage change (95% UE)
	Alcohol per capita (litres ethanol per year)	10.40	−1.64 (−2.04, −1.22)	−15.8 (−19.7, −11.8)	+0.93 (+0.63, +1.23)	+9.0 (+6.2, +11.8)
Harms (No. of cases, deaths, or years of life lost)	Mortality	3,846 (3,233, 4,420)	−834 (−1,306, −362)	−21.7 (−34.0, −9.4)	+562 (+57, +1,067)	+14.6 (+1.5, +27.7)
	PYPPLs^a^	15,664 (14,365, 16,788)	−3,966 (−5,078, −2,793)	−25.3 (−32.4, −17.8)	+2,389 (+1,289, +3,511)	+15.3 (+8.2, +22.4)
	Long-term disability cases	2,777 (2,733, 2,820)	−1,185 (−1,402, −946)	−42.7 (−51.3, −33.6)	+1,040 (+739, +1,405)	+37.5 (+26.6, +50.6)
	Hospital stays	44,020 (38,809, 49,038)	−15,420 (−19,727, −10,744)	−35.0 (−44.8, −24.4)	+11,836 (+5,534, +19,903)	+26.9 (+12.6, +45.2)
	Total criminal justice events^b^	270,652 (269,645, 271,531)	−59,687 (−102,824, −10,318)	−22.1 (−38.0, −3.8)	+37,590 (+9,569, +62,073)	+13.9 (+3.5, +22.9)
Costs (estimated economic cost in millions of Euros)	Economic loss of production due to PYPPLs	562.8 (516.3, 602.7)	−141.2 (−180.8, −99.4)	−25.1 (−32.1, −17.7)	+84.8 (+46.1, +124.2)	+15.1 (+8.2, +22.1)
	Long-term disability cases	115.1 (113.3, 116.9)	−49.2 (−58,2, −39.3)	−42.7 (−51.3, −33.6)	+43.2 (+30.7, +58.3)	+37.5 (+26.6, +50.6)
	Hospital stays	190.2 (162.3, 217.1)	−66.9 (−87.3, −45.0)	−35.2 (−45.9, −23.7)	+49.9 (+21.0, +85.1)	+26.2 (+11.0, +44.7)
	Total criminal justice costs	645.0 (642.6, 647.1)	−127.0 (−232.9, −5.8)	−19.7 (−36.1, −0.9)	+111.8 (+43.0, +171.9)	+17.3 (+6.7, +26.7)
	Total cost	1,513.1 (1,434.5, 1,583.7)	−384.3 (−559.2, −189.5)	−25.4 (−37.0, −12.5)	+289.7 (+140.8, +439.5)	+19.1 (+9.3, +29.0)

*Note.* PYPPL = potential years of productive life lost; UE = uncertainty estimate.

^a^Defined as years of life lost up to age 65 years. ^b^See Supplementary Table S5.

At baseline, alcohol was responsible for €1,513.1 million in economic costs. This cost was estimated to decrease by €384.3 million (or 25.4%) in scenario 1 and increase by €289.7 million (19.1%) in scenario 2; both estimates were significant (see Table 3).

In the baseline scenario, alcohol was estimated to have caused 3,846 deaths and 15,664 potential years of productive life lost (PYPPLs), resulting in €562.8 million lost productivity costs in 2018 (Table S1). In scenario 1, it was estimated that 834 deaths (−21.7% compared to baseline) and 3,966 PYPPLs (−25.3% compared to baseline) would be prevented, resulting in a reduction of €141.2 million, or 25.1% (95% CIs: −32.1%, −17.7%) in lost production. Scenario 2 led to an added 562 deaths (+14.6% compared to baseline) and €84.8 (€46.1, €124.2) million in lost production costs. In all scenarios, cardiovascular conditions were the leading cause of death, followed by digestive conditions (See Supplementary Tables S1–S4).

Table 2 depicts long-term disability (LTD) claims (2,777 at baseline) and hospital stays (44,020 at baseline). Relative to baseline, scenario 1 was estimated to prevent 1,185 LTD cases and 15,240 hospital stays, while scenario 2 was estimated to cause an additional 1,040 LTD cases and 11,836 hospital stays. All results were significant. A high proportion of alcohol-caused LTD cases were due to alcohol use disorders (90.3%) and resulting scenario changes were driven by this category.

There were an estimated 233,249 police-reported crime events, 27,187 court cases, and 10,216 corrections cases caused by alcohol in Finland in 2018; together these cost €645.0 million. In scenario 1, the total number and cost of these events would be reduced by an estimated 59,687 (10,318, 102,824) and €128.5 (€5.9, €235.7) million, respectively. Scenario 2 was estimated to lead to 13.9% more events and 17.3% more costs.

**Figure 1.**
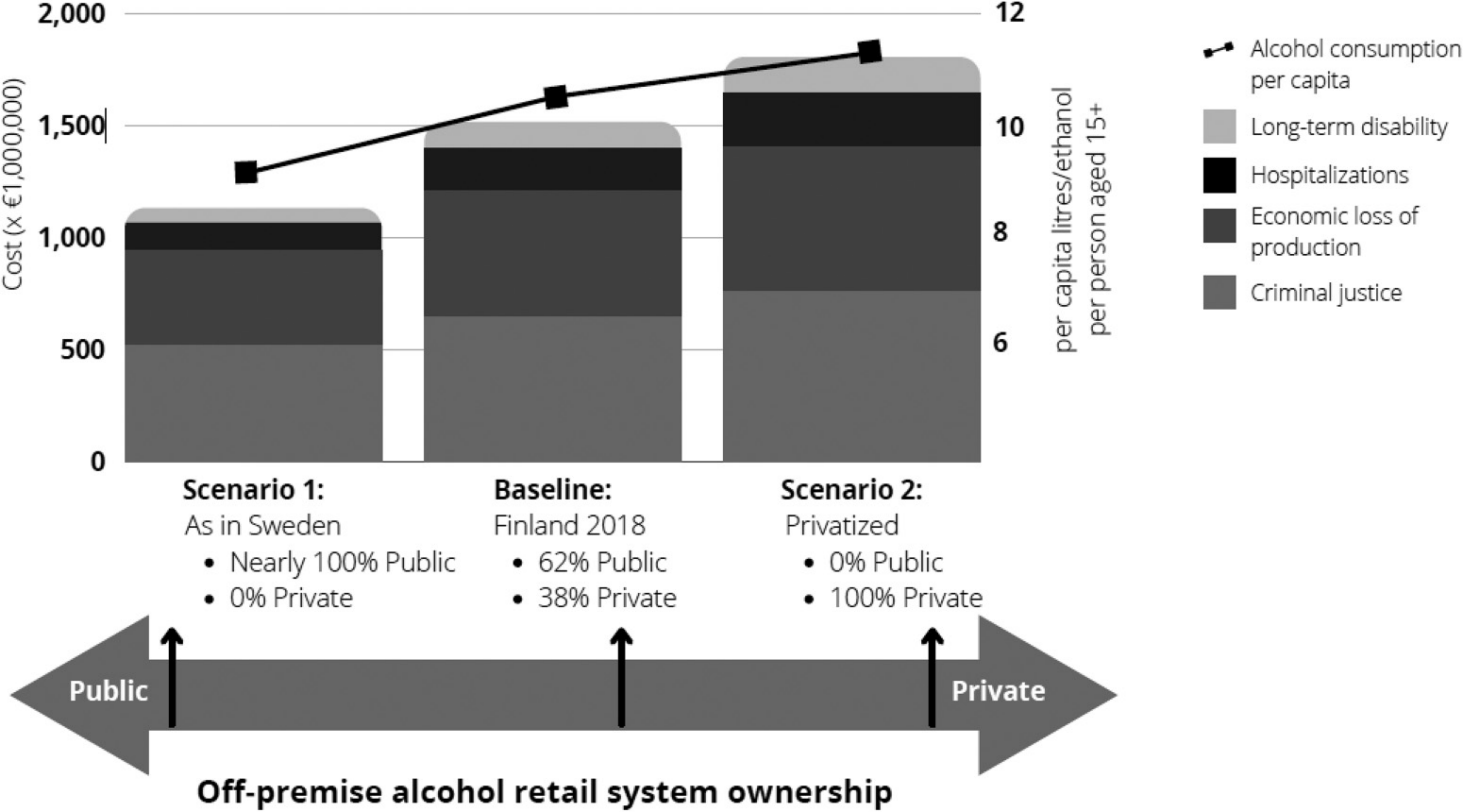
Summary figure showing total alcohol-caused cost and estimated alcohol use per capita, by alcohol retail ownership scenario, Finland, 2018.

Figure 1 depicts a summary of retail privatisation, showing the total costs and alcohol use per capita, as estimated by the current study.

## Discussion

Alcohol use causes substantial social cost and harm in Finland, and led to an estimated 3,846 deaths, 44,020 hospital stays, and 270,652 crime events and over €1.5 billion of economic costs in 2018 alone. These cost estimates are conservative, as they do not include the costs of alcohol-attributable drug prescriptions, specialised addictions treatment, emergency department visits, or day surgeries included in other alcohol costing studies (Canadian Substance Use Costs and Harms Scientific Working Group, 2018).

Alternative estimates of alcohol-caused harms were then estimated under two contrasting scenarios representing realistic policy choices regarding alcohol retailing in Finland. First, it was estimated that if Finland increased public ownership of the alcohol retail sales system to include all alcohol products with 3.5% ABV or higher, then alcohol use would be reduced by 15.8%. Conversely, a total privatisation of alcohol sales would result in a 9.0% increase in alcohol use. The scale of the resulting alcohol use per capita estimates under each scenario closely matches what is currently observed in other European countries, e.g., for Iceland and Sweden's public monopolies or UK and Switzerland privatised systems (European Commission, 2018). The estimated economic and health impacts of these changes in alcohol use per person is substantial. Expanding public ownership with resulting decreases in alcohol availability could have prevented 834 alcohol-caused deaths, 15,420 hospital stays, 59,687 crime events, and €384.3 million in 2018. By contrast, fully privatising alcohol sales could have resulted in increased harms and costs.

The alcohol use per capita and alcohol-attributable harm change estimates from the privatisation scenario for Finland are somewhat smaller than those previously estimated for privatisation in Sweden in previous studies (Norström et al., 2010; Stockwell et al., 2018). This is reasonable as Finland has to date privatised a larger fraction of alcohol sales than Sweden. Despite this, our estimates of harm and cost impacts of policy changes are conservative as they do not account for, for example, less effective restrictions on sales to underage customers or of advertising and marketing.

Among several limitations, InterMAHP estimates do not include harms experienced by those other than the drinker, e.g., alcohol-caused birth defects and child neglect (Sherk, Stockwell, Rehm, et al., 2020). It is also important to note the methodological assumption that the proposed policy changes in each scenario occurred far enough in the past for the impact of the resulting alcohol use changes to be reflected in the current incidence of chronic diseases caused by alcohol use.

In the context of alcohol retail, this study addresses an age-old decision facing governments around the world: are certain vital services better organised as public or private endeavours? Our results suggest that increased public ownership of the alcohol retail system is likely to lead to substantial improvements in population health and reduced economic costs at the expense of relatively minor reductions in affordability and convenience of access for consumers to a product with known health and safety risks. In addition, increased public ownership would increase public revenue. Conversely, further deregulation of alcohol markets risks increasing the economic burdens on the healthcare and criminal justice systems. Policymakers in Finland, and around the world, may reflect on the results of this study when considering the public or private organisation of their alcohol retail monopolies.

## Supplemental Material

sj-docx-1-nad-10.1177_14550725231160335 - Supplemental material for The public-private decision for alcohol retail systems: Examining the economic, health, and social impacts of alternative systems in Finland Click here for additional data file. Supplemental material, sj-docx-1-nad-10.1177_14550725231160335 for The public-private decision for alcohol retail systems: Examining the economic, health, and social impacts of alternative systems in Finland by Adam Sherk, Tim Stockwell, Justin Sorge, Samuel Churchill, Colin Angus, Tanya Chikritzhs, John Holmes, Petra Meier, Timothy S. Naimi, Thor Norström, Mats Ramstedt and Jussi Simpura in Nordic Studies on Alcohol and Drugs

## Appendix A1: THL costs of a hospital stay, by chapter

*Provided by special request to the Finnish Institute of Health and Welfare (THL)*

**Table:**

Year	ICD-10 chapter	Average cost (€)
2016	A00-B99	4,387
2016	C00-D49	5,940
2016	D50-D99	5,360
2016	E00-E99	4,041
2016	F00-F99	3,368
2016	G00-G99	4,152
2016	H00-H59	2,549
2016	H60-H99	3,292
2016	I00-I99	6,350
2016	J00-J99	3,761
2016	K00-K99	4,331
2016	L00-L99	4,078
2016	M00-M99	5,017
2016	N00-N99	3,546
2016	Q00-Q99	8,994
2016	S00-T99	4,963
2016	Other	3,421
2016	DG missing	3,150

## Appendix A2: Additional information regarding section 2.5 (Criminal justice: Policing, courts, and corrections)

*Criminal justice: Policing, courts, and corrections*.

Crime events were divided into four categories: homicide; other violent crimes; non-violent crime; and alcohol-defined crimes (such as drink driving). The alcohol-attributable fraction (AAF) for the last is 1.0. For the other categories, AAFs were derived as follows. For homicide, Granath et al. (2011) reported that 80.0% of homicide perpetrators were under the influence of alcohol in Finland. A Canadian study reported that 81.1% of inmates who were under the influence of alcohol at the time of their crime stated they would not have committed the crime if they had not been drinking (33). The AAF for homicide (0.800*0.811 = 0.649) is therefore a two-factor adjustment. An AAF of 0.608 for assault was derived from a Finnish study (34). An AAF of 0.244 for non-violent crimes was derived for Finland from the Canadian Substance Use Costs and Harms study assuming a fixed ratio to violent crime (27). These category-level AAFs were then combined by weighting by the number of police-reported events, court cases, and correction cases. Weighted AAFs were applied to the aggregated cost data for each outcome.

**Table A2.1.** Finnish crime alcohol AFs by crime group.

Crime group	Alcohol AF
Homicide	0.6488
Other violent crimes	0.6080
Non-violent crimes	0.2440
Alcohol-specific crimes	1.000
