# Supplementary material for: The public-private decision for alcohol retail systems: Examining the economic, health, and social impacts of alternative systems in Finland
Source: Nordisk Alkohol Nark. 2023 Mar 15;40(3):218–32. doi: 10.1177/14550725231160335 (PMC10225965; doi:10.1177/14550725231160335)
Supplement: sj-docx-1-nad-10.1177_14550725231160335 - Supplemental material for The public-private decision for alcohol retail systems: Examining the economic, health, and social impacts of alternative systems in Finland [file sj-docx-1-nad-10.1177_14550725231160335.docx]

Supplementary Table S1: Estimated levels at baseline and changes under two alcohol retail system scenarios of alcohol-attributable **mortality** counts (by health condition category), **potential years of productive life lost** and the **cost of economic loss of production** in Finland, 2018

| **Health Condition Category** | **Baseline**  Finland in 2018 | **Scenario 1: More Public Ownership**  (Similar to Sweden) | | **Scenario 2: More Private Ownership**  (All alcohol sold in grocery stores) | |
| --- | --- | --- | --- | --- | --- |
|  | **Estimate**  (95% UE) | **Estimated Change**  (95% UE) | **Percentage Change**  (95% UE) | **Estimated Change**  (95% UE) | **Percentage Change**  (95% UE) |
| Communicable Diseases | **30**  (24; 37) | **-6**  (-7; -4) | **-18.1%**  (-24.1%; -11.5%) | **+3**  (+1; +5) | **+10.7%**  (+4.2%; +17.6%) |
| Cancers | **501**  (426; 572) | **-63**  (-108; -18) | **-12.5%**  (-21.5%; -3.5%) | **+37**  (-8; +81) | **+7.3%**  (-1.6%; +16.1%) |
| Diabetes | **-16**  (-20; -12) | **+1**  (-2; +4) | * | **0**  (-4; +3) | * |
| Neuropsychiatric Conditions | **306**  (303; 309) | **-130**  (-155; -102) | **-42.6%**  (-50.5%; -33.4%) | **+108**  (+70; +151) | **+35.2%**  (+22.8%; 49.5%) |
| Cardiovascular Conditions | **1,363**  (980; 1,736) | **-220**  (-504; 55) | **-16.1%**  (-37.0%; +4.0%) | **+151**  (-140; +434) | **+11.1%**  (-10.3%; 31.8%) |
| Digestive Conditions | **855**  (773; 916) | **-95**  (-248; -139) | **-22.8%**  (-29.0%; -16.3%) | **+123**  (+73; +176) | **+14.4%**  (+8.5%; +20.5%) |
| Motor Vehicle Collisions | **57**  (52; 62) | **-15**  (-20; -11) | **-26.7%**  (-34.3%; -18.5%) | **+9**  (+4; +14) | **+15.6%**  (+7.3%; +24.0%) |
| Unintentional  Injuries | **526**  (495; 557) | **-140**  (-177; -101) | **-26.6%**  (-33.7%; -19.1%) | **+89**  (+44; +137) | **+17.0%**  (+8.4%; +26.0%) |
| Intentional Injuries | **223**  (201; 244) | **-66**  (-84; -46) | **-29.6%**  (-37.9%; -20.6%) | **+41**  (+17; +67) | **+18.5%**  (+7.4%; +29.9%) |
| ***TOTAL DEATHS*** | ***3,846***  *(3,233; 4,420)* | ***-834***  *(-1,306; -362)* | ***-21.7%***  *(-34.0%; -9.4%)* | ***+562***  *(+57; +1,067)* | ***+14.6%***  *(+1.5%; +27.7%)* |
| ***TOTAL Potential Years of Productive Life Lost (PYPLLs)*** | ***15,664***  *(14,365; 16,788)* | ***-3,966***  *(-5,078; -2,793)* | ***-25.3%***  *(-32.4%; -17.8%)* | ***+2,389***  *(+1,289; +3,511)* | ***+15.3%***  *(+8.2%; +22.4%)* |
| ***TOTAL COST of economic loss of production***  ***(Millions of Euros)*** | ***562.8***  *(516.3; 602.7)* | ***-141.2***  *(-180.8; -99.4)* | ***-25.3%***  *(-32.1%; -17.7%)* | ***+84.8***  *(+46.1; +124.2)* | ***+15.1%***  *(+8.2%; +22.1%)* |

Column totals may not add due to rounding; *These percentages are not applicable.

Supplementary Table S2: Estimated levels at baseline and changes under two alcohol retail system scenarios of alcohol-attributable **long-term disability** counts (by ICD10 chapter) and total cost in Finland, 2018

| **ICD10 Chapter** | **Baseline**  Finland in 2018 | **Scenario 1: More Public Ownership**  (Similar to Sweden) | | **Scenario 2: More Private Ownership**  (All alcohol sold in grocery stores) | |
| --- | --- | --- | --- | --- | --- |
|  | **Estimate**  (95% UE) | **Estimated Change**  (95% UE) | **Percentage Change**  (95% UE) | **Estimated Change**  (95% UE) | **Percentage Change**  (95% UE) |
| Neuropsychiatric conditions  (F00-F99) | **2,508**  (n/a) | **-1,122**  (-1,318; -904) | **-44.7%**  (-52.6%; -36.1%) | **+1,000**  (+720; +1,342) | **+37.5%**  (+28.7%; +53.5%) |
| Diseases of the nervous system  (G00-G99) | **144**  (121; 166) | **-28**  (-36; -20) | **-19.5%**  (-30.0%; -11.8%) | **+17**  (+9; +25) | **+11.7%**  (+6.2%; +17.2%) |
| Injuries and poisonings  (S00-T99) | **61**  (54; 68) | **-19**  (-23; -13) | **-30.5%**  (-43.1%; -19.8%) | **+12**  (5; 20) | **+20.4%**  (+8.3%; 33.0%) |
| All other | **64**  (50; 78) | **-17**  (-24; -9) | **-26.5%**  (-49.0%; -11.9%) | **+11**  (+5; +17) | **+17.2%**  (+7.7%; +27.3%) |
| ***TOTAL COUNT*** | ***2,777***  *(2,733; 2,820)* | ***-1,185***  *(-1,402; -946)* | ***-42.7%***  *(-51.3%; -33.6%)* | ***+1,040***  *(+739; +1,405)* | ***+37.5%***  *(+26.6%; +50.6%)* |
|  |  |  |  |  |  |
| ***TOTAL COST (Millions of Euros)*** | ***115.1***  ***(113.3; 116.9)*** | ***-49.2***  *(-58.2; -39.3)* | ***-42.7%***  *(-51.3%; -33.6%)* | ***+43.2***  *(+30.7; 58.3%)* | ***+37.5%***  *(+26.6%; +50.6%)* |

Column totals may not add due to rounding

All other chapters include cancers, diabetes, cardiovascular conditions, respiratory conditions and digestive conditions.

*These percentages are not applicable.

Supplementary Table S3: Estimated levels at baseline and changes under two alcohol retail system scenarios of alcohol-attributable **hospital stay** counts (by health condition category) and total cost in Finland, 2018

| **Health Condition Category** | **Baseline**  Finland in 2018 | **Scenario 1: More Public Ownership**  (Similar to Sweden) | | **Scenario 2: More Private Ownership**  (All alcohol sold in grocery stores) | |
| --- | --- | --- | --- | --- | --- |
|  | **Estimate**  (95% UE) | **Estimated Change**  (95% UE) | **Percentage Change**  (95% UE) | **Estimated Change**  (95% UE) | **Percentage Change**  (95% UE) |
| Communicable Diseases | **3,633**  (2,914; 4,338) | **-626**  (-835; -404) | **-17.2%**  (-23.0%; -11.1%) | **+365**  (+148; +590) | **+10.1%**  (+4.1%; +16.3%) |
| Cancers | **3,109**  (2,625; 3,563) | **-449**  (-701; -197) | **-14.4%**  (-22.5%; -6.3%) | **+266**  (+15; +514) | **+8.6%**  (+0.5%; +16.5%) |
| Diabetes | **-174**  (-220; -130) | **+6**  (-26; 38) | * | **-3**  (-37; +30) | * |
| Neuropsychiatric Conditions | **18,429**  (18,178; 18,667) | **-7,855**  (-9,402; -6,115) | **-42.6%**  (-51.0%; -33.2%) | **+6,915**  (+4,255; +11,231) | **+37.5%**  (+23.1%; +60.9%) |
| Cardiovascular Conditions | **-1,084**  (-2,580; 389) | **-1,086**  (-1,818; -342) | * | **+732**  (-95; +1,558) | * |
| Digestive Conditions | **4,934**  (4,534; 5,305) | **-784**  (-1,081; -488) | **-15.9%**  (-21.9%; -9.9%) | **+461**  (+202; +751) | **+9.3%**  (+4.1%; +14.5%) |
| Motor Vehicle Collisions | **676**  (612; 737) | **-187**  (-239; -130) | **-27.6%**  (-35.4%; -19.3%) | **+110**  (+52; +170) | **+16.3%**  (+7.8%; +25.1%) |
| Unintentional  Injuries | **13,312**  (11,683; 14,889) | **-4,086**  (-5,172; -2,859) | **-30.7%**  (-38.8%; -21.5%) | **+2,770**  (+903; +4,745) | **+20.8%**  (+6.8%; +35.6%) |
| Intentional Injuries | **1,176**  (1,064; 1,281) | **-354**  (-453; -247) | **-30.1%**  (-38.5%; -21.0%) | **+219**  (+91; +350) | **+18.6%**  (+7.8%; +29.8%) |
| ***TOTAL COUNT*** | ***44,020***  *(38,809; 49,038)* | ***-15,420***  *(-19,727; -10,744)* | ***-35.0%***  *(-44.8%; -24.4%)* | ***+11,836***  *(+5,534; +19,903)* | ***+26.9%***  *(+12.6%; +45.2%)* |
|  |  |  |  |  |  |
| ***TOTAL COST (Millions of Euros)*** | ***190.2***  ***(162.3; 217.1)*** | ***-66.9***  *(-87.3; -45.0)* | ***-35.2%***  *(-45.9%; -23.7%)* | ***+49.9***  *(+21.0; +85.1)* | ***+26.2%***  *(+11.0%; +44.7%)* |

Column totals may not add due to rounding

*These percentages are not applicable.

Supplementary Table S4: Estimated levels at baseline and changes under two alcohol retail system scenarios of alcohol-attributable **criminal justice** counts and costs in Finland, 2018

|  | **Category** | **Baseline**  Finland in 2018 | **Scenario 1: More Public Ownership**  (Similar to Sweden) | | **Scenario 2: More Private Ownership**  (All alcohol sold in grocery stores) | |
| --- | --- | --- | --- | --- | --- | --- |
|  |  | **Estimate**  (95% UE) | **Estimated Change**  (95% UE) | **Percentage Change**  (95% UE) | **Estimated Change**  (95% UE) | **Percentage Change**  (95% UE) |
| Harms (Number of cases, deaths, or years of life lost) | Police-reported crime events | **233,248**  (232,381; 234,006) | -**51,438**  (-88,614; -8,892) | **-22.1%**  (-38.0%; -3.8%) | **+32,395**  (+8,247; +53,494) | **+13.9%**  (+3.5%; +22.9%) |
|  | Court cases | **27,187**  (27,086; 27,276) | **-5,996**  (-10,329; -1,036) | **-22.1%**  (-38.0%; -3.8%) | **+3,776**  (+961; + 6,235) | **+13.9%**  (+3.5%; +22.9%) |
|  | Corrections cases | **10,216**  (10,178; 10,249) | -**2,253**  (-3,881; -389) | **-22.1%**  (-38.0%; -3.8%) | **+1,419**  (+361; +2,343) | **+13.9%**  (+3.5%; +22.9%) |
|  | ***TOTAL EVENTS*** | ***270,652***  *(269,645; 271,531)* | ***-59,687***  *(-102,824; -10,318)* | ***-22.1%***  *(-38.0%; -3.8%)* | ***+37,590***  *(+9,569; +62,073)* | ***+13.9%***  *(+3.5%; +22.9%)* |
| Costs (Estimated economic cost in MM Euros) | Police-reported crime events | **357.2**  (355.9; 358.4) | **-70.3**  (-129.0; -3.2) | **-19.7%**  (-36.1%; -0.9%) | **+61.9**  (+23.8; +95.2) | **+17.3%**  (+6.7%; 26.7%) |
|  | Court cases | **173.0**  (172.3; 173.5) | **-34.1**  (-62.5; -1.6) | **-19.7%**  (-36.1%; -0.9%) | **+30.0**  (+11.5; +46.1) | **+17.3%**  (+6.7%; 26.7%) |
|  | Corrections cases | **114.8**  (114.4; 115.2) | **-22.6**  (-41.4; -1.0) | **-19.7%**  (-36.1%; -0.9%) | **+19.9**  (+7.7; +30.6) | **+17.3%**  (+6.7%; 26.7%) |
|  | ***TOTAL COST*** | ***645.0***  *(642.6; 647.1)* | ***-128.5***  *(-235.7, -5.9)* | ***-19.7%***  *(-36.1%; -0.9%)* | ***+111.8***  *(+43.0; +171.9)* | ***+17.3%***  *(+6.7%; 26.7%)* |

*Column totals may not add due to rounding
